# Correlates of burnout among healthcare workers during the COVID-19 pandemic in South Korea

**DOI:** 10.1038/s41598-023-30372-x

**Published:** 2023-02-27

**Authors:** Soyoon Hwang, Ki Tae Kwon, So Hee Lee, Shin-Woo Kim, Hyun-Ha Chang, Yoonjung Kim, Sohyun Bae, Hae Suk Cheong, Se Yoon Park, Bongyoung Kim, Shinwon Lee, Jiho Park, Sang Taek Heo, Won Sup Oh, Yeonjae Kim, Kyung-Hwa Park, Chang Kyung Kang, NamHee Oh, Su Jin Lim, Seongcheol Yun, Ji Woong Son, Hyun Wook Ryoo, Un Sun Chung, Ju-Yeon Lee, Hye Yoon Park, Ji-Yeon Shin, Sang-geun Bae, Ji-yeon Lee

**Affiliations:** 1 Division of Infectious Diseases, Department of Internal Medicine, Kyungpook National University Chilgok Hospital, School of Medicine, Kyungpook National University, 807 Hokuk-ro, Buk-gu, Daegu, 41404 South Korea; 2 Department of Psychiatry, National Medical Center, 245 Eulji-ro, Jung-gu, Seoul, 04564 South Korea; 3 Division of Infectious Diseases, Department of Internal Medicine, School of Medicine, Kyungpook National University Hospital, Kyungpook National University, Daegu, South Korea; 4 Division of Infectious Diseases, Department of Internal Medicine, Kangbuk Samsung Hospital, Sungkyunkwan University School of Medicine, Seoul, South Korea; 5 Division of Infectious Diseases, Department of Internal Medicine, Soonchunhyang University Seoul Hospital, Soonchunhyang University College of Medicine, Seoul, South Korea; 6 Department of Internal Medicine, Hanyang University College of Medicine, Seoul, South Korea; 7 Department of Internal Medicine, Pusan National University School of Medicine and Medical Research Institute, Pusan National University Hospital, Busan, South Korea; 8 Department of Internal Medicine, Konkuk University School of Medicine, Konkuk University Medical Center, Seoul, South Korea; 9 Division of Infectious Diseases, Department of Internal Medicine, College of Medicine, Jeju National University, Jeju, South Korea; 10 Division of Infectious Diseases, Department of Internal Medicine, Kangwon National University School of Medicine, Chuncheon, South Korea; 11 Division of Infectious Disease, Department of Internal Medicine, National Medical Center, Seoul, South Korea; 12 Department of Infectious Diseases, Chonnam National University Medical School, Gwangju, South Korea; 13 Department of Internal Medicine, Seoul National University College of Medicine, Seoul, South Korea; 14 Hospital Infection Control Team, Daegu Medical Center, Daegu, South Korea; 15 Division of Respiratory Diseases, Department of Internal Medicine, Masan Medical Center, Changwon, South Korea; 16 Department of Internal Medicine, Andong Medical Center, Andong, South Korea; 17 Department of Internal Medicine, Konyang University Hospital, Daejeon, South Korea; 18 Department of Emergency Medicine, School of Medicine, Kyungpook National University, Daegu, South Korea; 19 Department of Psychiatry, School of Medicine, Kyungpook National University, Daegu, South Korea; 20 Department of Psychiatry, Chonnam National University Medical School, Gwangju, South Korea; 21 Department of Psychiatry, Seoul National University Hospital, Seoul, South Korea; 22 Department of Preventive Medicine, School of Medicine, Kyungpook National University, Daegu, South Korea; 23 Department of Counseling Psychology, Hankuk University of Foreign Studies, Seoul, South Korea

**Keywords:** Psychology, Diseases, Health care, Health occupations, Risk factors

## Abstract

Burnout is a form of negative emotional and physical response to job stress. This study aimed to investigate the prevalence of burnout among healthcare workers responding to the coronavirus disease 2019 (COVID-19) outbreak in Korea and to explore correlates of burnout among healthcare workers. A nationwide questionnaire-based survey was conducted from December 1, 2020, to January 29, 2021 on 1425 healthcare workers who worked in one of the 16 healthcare facilities designated for COVID-19 care, in public health centers, or as paramedics in Korea. Burnout was assessed using 16 Korean-adapted items based on the Oldenburg Burnout Inventory (OLBI). Data were collected using a structured questionnaire and analyzed using the R version 4.1.1 software program. OLBI results indicate clinically exhaustion in 84.5% (1204/1425) and clinically disengagement in 91.1% (1298/1425), and 77.3% (1102/1425) met the score criteria for both the exhaustion and disengagement subscales for burnout. Burnout rate was significantly increased in the group with chronic fatigue symptoms (Fatigue Severity Scale ≥ 3.22) after the outbreak of COVID-19 (OR, 3.94; 95% CI 2.80–5.56), in the female group (OR, 2.05; 95% CI 1.46–2.86), in the group with physical symptoms (Patient Health Questionnaire-15 ≥ 10) after the outbreak of COVID-19 (OR, 2.03; 95% CI 1.14–3.60), in the group with a higher Global Assessment of Recent Stress scale (OR, 1.71; 95% CI 1.46–2.01), in the group with post-traumatic stress symptoms (Primary Care Post-Traumatic Stress Disorder-5 ≥ 2) (OR, 1.47; 95% CI 1.08–2.01), and in the younger age group(OR, 1.45; 95% CI 1.22–1.72). The chronic fatigue symptoms were correlated with cumulative days of care (OR, 1.18; 95% CI 1.02–1.37). The physical symptoms were correlated with average contact hours with COVID-19 patients per day (OR, 1.34; 95% CI 1.17–1.54), and cumulative days of care (OR, 1.21; 95% CI 1.06–1.38). Most Korean healthcare workers suffered from burnout related to excessive workload during the COVID-19 pandemic. During a widespread health crisis like COVID-19, it is necessary to regularly check the burnout status in healthcare workers and reduce their excessive workload by supplementing the workforce and providing appropriate working hours sufficient rest hours.

## Introduction

The World Health Organization (WHO) declared the coronavirus disease 2019 (COVID-19) as a pandemic on March 11, 2020; subsequently, more than 300 million confirmed cases of COVID-19 were reported worldwide by January 1, 2022^1,2^. With the prolonged nature of the COVID-19 pandemic, across the globe, healthcare workers involved in COVID-19 care have hit their physical and mental limits. A healthcare system’s collapse due to a pandemic caused by a novel infectious disease, such as COVID-19, can expose healthcare workers to stress. During the H1N1 pandemic, nurses involved in patient care developed mental health conditions, such as stress, anxiety, depression, and hostility. They avoided being involved in caring for patients with the infectious disease due to the fear of exposing themselves or their families to the virus and the consequent mental stress^3^. During the severe acute respiratory syndrome (SARS) pandemic, nurses caring for patients with SARS had higher levels of stress due to the use of personal protection equipment (PPE), risk of virus exposure, infectious disease management, infection management protocol, and patients’ demands^4^. As the COVID-19 pandemic continues, studies on depression, anxiety, and stress of healthcare workers related to COVID-19 have been conducted in several countries^5–8^. Since these workers often face novel and difficult situations, various symptoms ranging from psychological distress to mental disorders can occur, and policies to prevent these symptoms are urgently needed^9,10^.

“Burnout” is a form of negative emotional and physical response to job stress that was first described in 1974 by a German American psychologist named Freudenberger^11^. It can occur when members of an organization do not receive the expected reward or can no longer manage their stress due to excessively committing to interpersonal relationships in the workplace. The Job demands-resources (JD-R) model suggests that work-related burnout progresses through two mechanisms. The first one is related to ‘exhaustion’ from excessive job demands and the second one is related to ‘disengagement’ from lack of job resources^12^. JD-R model proposes job demands, such as high workload, time pressure, and emotional demands, may initiate the processes of losing energy and impairing health, which in turn lead to chronic exhaustion and burnout^12,13^. During an outbreak of a novel infectious disease such as COVID-19, healthcare workers may be exposed to exhaustion and burnout due to increased job demands such as staff shortage and complicated protocols^14^. A high level of burnout among healthcare staff involved in COVID-19 care has already been documented in multiple countries that have experienced a collapse of the healthcare system due to the COVID-19 pandemic, including the United States, India, Italy, the United Kingdom, and Singapore^15–19^. The burnout of healthcare workers can negatively affect their relationship with patients, reduce the quality of medical services, and negatively affect their personal lives, such as turnover^20^.

At the beginning of the COVID-19 pandemic, South Korea’s strong national response to COVID-19 provided several important lessons for other countries^21,22^. The Korean government designated hospitals for the care of COVID-19 patients centered on public hospitals, and quickly created negative-pressure isolation rooms using portable negative-pressure devices so that all patients diagnosed with COVID-19 at the pandemic could be hospitalized^21^. The government tried to recruit unpaid volunteers, and hospitals recruited new employees, but it was difficult to solve the shortage of healthcare workers. The excessive workload required for the success of containment measures has led to the burnout of healthcare workers^23^. This study is the first large-scale nationwide study conducted in Korea to determine the prevalence of burnout in healthcare workers during the COVID-19 pandemic and analyze predictive factors for it.

## Method

### Participants and sample size

The study population comprised physicians, nurses, other healthcare workers, public health center staff, and epidemiologists, who worked in one of the 16 healthcare facilities designated for COVID-19 care and public emergency medical service workers between January 20, 2020, and December 1, 2020. Table 1 is a list of participating medical institutions nationwide.

**Table 1:** List of participating medical institutions by region.

City or Province	Number	Name of Medical Institution
Seoul Metropolitan City	6	Gangbuk Samsung Hospital
		Hanyang University Seoul Hospital
		Konkuk University Hospital
		SoonChunHyang University Seoul Hospital
		Seoul National University Hospital
		National Medical Center
Daegu Metropolitan City	3	Kyongpook National University Hospital
		Kyongpook National University Chilgok Hospital
		Daegu Medical Center
Busan Metropolitan City	1	Pusan National University Hospital
Daejeon Metropolitan City	1	Konyang University Hospital
Gwangju Metropolitan City	1	Chonnam National University Hospital
Gangwon Province	1	Kangwon National University Hospital
Gyeongsangbuk Province	1	Andong Medical Center
Gyeongsangnam Province	1	Masan Medical Center
Jeju Special Self-Governing Province	1	Jeju National University Hospital

**Figure 1:**
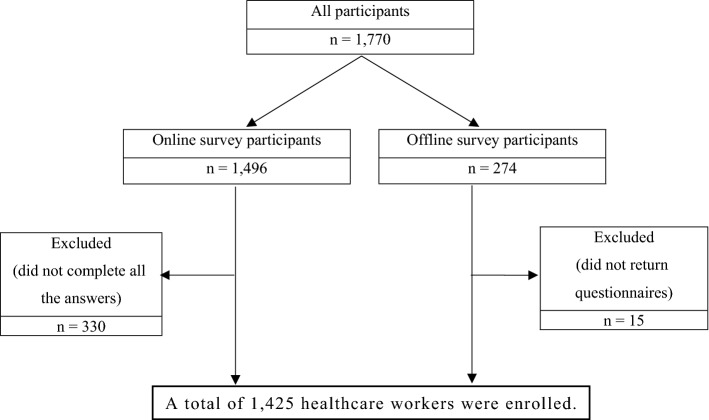
Flow chart describes the study enrollment steps.

Considering the sampling accuracy and economic cost of increasing the sample size, the optimal population sample size was approximately 1000–1500 people^24^. The target sample size for each job group was determined to be 150 physicians, 700 nurses, 300 other workers (e.g., clinical pathologists, radiologic technologists, cleaning staff, and administrative staff) in consideration of the number of healthcare workers involved in COVID-19-related work. In the case of emergency medical service workers working outside the healthcare facilities, about 300 people were set as the target sample, and with the help of the National Emergency Management Agency, an online survey website link was sent through a message. Finally, 1166 of 1496 people who participated in the online survey completed all the answers, and 259 of 274 people who received the offline questionnaire completed and returned it, resulting in a total 1425 responses analyzed (Fig. 1).

### Study design

A text message with a link to an online survey web site was sent to potential candidates who worked in one of the 16 healthcare facilities designated for COVID-19 care, public health centers, or emergency medical services in Korea from December 1, 2020, to January 29, 2021. During the same period, a paper-and-pencil survey was sent by mail to those candidates who wanted an offline survey. The online questionnaire was accessed via the SurveyMonkey link, and those who read the information page and consented to participate were allowed to proceed with the questionnaire. The offline questionnaire was distributed along with a study consent form, and the completed questionnaires were collected either in person or via mail.

### Measures

An supplementary note shows the questionnaire that was developed with items about the physical and mental impact of COVID-19 care during the COVID-19 pandemic [see Supplementary Note file]. Sociodemographic factors included gender, age, occupation, work location, and length of employment. For healthcare workers involved in COVID-19 care, the following were additionally surveyed: information about the number of confirmed patients they had provided care for, duration of COVID-19 care, duration of contact with COVID-19 patients, experiences with especially difficult patients, such as those critically or mentally ill, use of PPE, exposure to infection risk, and training for wearing and removing PPE. Physical and mental health assessment included instruments to assess perceived physical symptoms, chronic fatigue symptoms, post-traumatic stress disorder (PTSD) symptoms, depression symptoms, anxiety disorder, sleep disorder, burnout, perceive stress, positive resources, job satisfaction, and intent to practice nursing or turnover upon another outbreak of a novel infectious disease. The following scales were selected in reference to several previous studies on effective factors that determine the prevalence of burnout in healthcare workers^6,10,18–20^. In particular, the positive resource scale using the Positive Resources Test (POREST) was a protective factor in a previous study on burnout during the MERS epidemic in Korea^25^, but there are few studies on the positive resource and burnout. Therefore, we tried to investigate the effect of positive resources on burnout using POREST in our study.

Perceived physical symptoms were assessed using the Patient Health Questionnaire-15 (PHQ-15), with 0 for “none,” 1 for “mild,” and 2 for “very severe.” A total score of 10 or higher indicated moderate physical symptoms^26^. Cronbach’s α was 0.80 in Kroenke et al.’s study^26^, and in this study, it was 0.82 for questions before COVID-19 and 0.88 for questions after COVID-19.

Chronic fatigue symptoms were assessed using the nine-item Fatigue Severity Scale (FSS). Each item was rated on a seven-point scale, and an average score of 3.22 or higher indicated fatigue^27^. Cronbach’s α was 0.93 in Krupp et al.’s study^28^, and in this study, it was 0.93 for questions before COVID-19 and 0.94 for questions after COVID-19.

PTSD symptoms were assessed using the Primary Care Post-Traumatic Stress Disorder-5 (PC-PTSD-5), with 0 for “no” and 1 for “yes.” A score of 2 was considered “moderate PTSD,” and a score of 3 or higher was considered “severe PTSD”^29^. In Jung et al.’s study, Cronbach’s α was 0.87^29^, and it was. 76 in this study.

Depression symptoms were assessed using the Korean version of the Patient Health Questionnaire-9 (PHQ-9), which asks about the frequency of symptoms in the previous two weeks using a scale with 0 for “none,” 1 for “two days or more,” 2 for “one week or longer,” and 3 for “almost every day.” A total score of 10 or higher was defined as “moderate or more severe depression symptoms”^30,31^. In Spitzer et al.’s study, Cronbach’s α was 0.94^32^, and in this study, it was 0.87 for the question before COVID-19 and 0.89 for the question after COVID-19.

Anxiety symptoms were assessed using the Generalized Anxiety Disorder Scale (GAD-7), with the same scoring criteria as that for depression (0–3). A score of 10 or higher was defined as “moderate or more severe anxiety symptoms”^33,34^. In Spitzer et al.’s study, Cronbach’s α was 0.92^35^, and in this study, it was 0.89 for both pre- and post-COVID-19 questions.

Insomnia was assessed using seven items in the Korean-adapted version (2013) of the Insomnia Severity Index developed by Bastien et al.^36^. Each item was rated on a 0–4 scale, and a total score of 16 or higher was defined as insomnia^36,37^. In Cho et al.^38^ Cronbach’s α was 0.92, and in this study it was 0.92 as well.

Stress was assessed using the Global Assessment of Recent Stress (GARS) scale, which measures perceived stressors in the previous week. Each of the eight items about work, school life, interpersonal relationships, changes in relationship, disease and injury, economic problems, unordinary events, changes in daily life, and overall perceived stress was rated on a scale from 0 (no stress at all) to 9 (extreme stress). A higher score indicated more significant perceived stress^39^. In Koh et al.’s study, Cronbach’s α was 0.86^39^, and in this study, it was 0.89.

Positive resources were assessed using the POREST developed by Kim et al. in 2013. The 23-item test comprised seven items for positivity, six for purpose and hope, five for self-control, three for social support, and two for caregiving and service. Each item was rated on a 1–5 scale, and the total score range was 23–115, where a higher score indicated greater positive resources^40,41^. In Chae et al.’s study, Cronbach’s α was 0.92^42^, and in this study, it was 0.92 as well.

Burnout was assessed using 16 Korean-adapted items by Na based on the Oldenburg Burnout Inventory (OLBI) developed by Demerouti, which comprises eight items for exhaustion and eight items for disengagement^43–45^. Each items rated on a 4-point Likert scale with options of “Strongly disagree,” “Disagree,” “Agree,” and “Strong agree,” and the burnout response is the highest with 4 points and the lowest with 1 point. Eight of the items are reverse-scored. The means were calculated for each items for two domains, exhaustion and disengagement. Burnout was determined with a cutoff score of 2.25 for exhaustion and 2.10 or higher for disengagement^43^. In Peterson’s study, Cronbach’s α was. 83^43^, and in this study, it was 0.90.

### Data analysis

All analyses were conducted using the R version 4.1.1 software. The participants’ demographics and information assessing burnout were summarized using standard descriptive statistics. Descriptive statistics were also used to compare the burnout rate of groups according to demographic characteristics, presence of physical and psychological symptoms, and workload related COVID-19. Univariate logistic analysis of participants’ characteristics and information was performed to evaluate the predictive factors of burnout. The differences in categorical variables other than the stress and positive resources scores were analyzed using Pearson’s Chi-square test and Fisher’s exact test. The differences in the continuous variables (stress score and positive resources score) were analyzed using student t-tests or a Wilcoxon test. All factors with a *p*-value of less than 0.05 in univariate analysis were placed in a multivariable logistic regression analysis using a stepwise approach to investigate predictors of burnout. We also reported the receiver operating characteristic (ROC) curve of statistically significant variables after multivariable logistic regression analysis and calculate the area under curve (AUC) to evaluate the sensitivity and specificity of this logistic regression model in predicting the burnout. A *p*-value of less than 0.05 was interpreted as significant. Due to concerns about the validity of the analysis of burnout as categorical variable, correlations between the total score of each of the two domains of burnout and clinical variables were examined using Pearson’s r and Spearman’s rho. Multiple linear regression was used to control confounding variables and identify the predictors of higher burnout scores.

### Institutional review board statement

The study was approved by the Public Institutional Review Board Designated by Ministry of Health and Welfare, Seoul, South Korea (IRB number: P-01-202011-23-001). Written informed consent was obtained from each participant who volunteered to participate after reading the information about the study. All procedures performed in studies involving human participants were in accordance with the ethical standards of the institutional and/or national research committee and with the 1964 Helsinki Declaration and its later amendments or comparable ethical standards.

## Results

### Burnout according to the demographic characteristics

Table 2 depicts the demographic characteristics and burnout rate of the participants. Among the 1,425 participants, 923 were women (64.8%), and more than 80% of the participants were aged 20–29 (n = 581, 40.8%) and 30–39 (n = 565, 39.6%) years. Most participants (n = 712, 50%) were nurses, and the majority worked in a university hospital (n = 741, 52.0%). The most common employment period was one to five years (n = 686, 48.1%), followed by six to ten years (n = 279, 19.6%), and less than one year (n = 175, 12.3%).

**Table 2:** Characteristics of the study population and burnout rate according to the characteristics.

Variable	Total (N = 1425)	Burnout (N = 1102)	No Burnout (N = 323)	*P*-value
Gender				*P* < 0.001
Men	502	309 (61.6%)	193 (38.4%)	
Women	923	793 (85.9%)	130 (14.1%)	
Age group				*P* < 0.001
20–29 years	581	480 (82.6%)	101 (17.4%)	
30–39 years	565	440 (77.9%)	125 (22.1%)	
40–49 years	173	123 (71.1%)	50 (28.9%)	
50–59 years	90	51 (56.7%)	39 (43.3%)	
> 60 years	16	8 (50.0%)	8 (50.0%)	
Current region of work				*P* < 0.001
Seoul metropolitan area	704	545 (77.4%)	159 (22.6%)	
Gangwon	47	38 (80.9%)	9 (19.1%)	
Chungcheong	110	82 (74.5%)	28 (25.5%)	
Jeolla	71	31 (43.7%)	40 (56.3%)	
Gyeongsang	485	400 (82.5%)	85 (17.5%)	
Jeju	8	6 (75%)	2 (25%)	
Type of facility				*P* < 0.001
National/public medical center	320	254 (79.4%)	66 (20.6%)	
University hospital (Tertiary hospital)	741	614 (82.9%)	127 (17.1%)	
Public emergency medical service	326	203 (62.3%)	123 (37.7%)	
Others	38	31 (81.6%)	7 (18.4%)	
Length of current employment				0.018
< 1 year	175	121 (69.1%)	54 (30.9%)	
1–5 years	686	544 (79.3%)	142 (20.7%)	
6–10 years	279	223 (79.9%)	56 (20.1%)	
> 10 years	285	214 (75.1%)	71 (24.9%)	
Occupation/profession				*P* < 0.001
Physician	167	128 (76.6%)	39 (23.4%)	
Nurse	712	633 (88.9%)	79 (11.1%)	
Paramedic	297	184 (62.0%)	113 (38.0%)	
Nurse aid, transport staff, cleaning staff, and radiologic technologist	135	77 (57.0%)	58 (43.0%)	
Hospital administrative staff and others	114	80 (70.2%)	34 (29.8%)	

A total of 1204 (84.5%) participants had an exhaustion score of 2.25 or higher, and 1,298 (91.1%) had a disengagement score of 2.1 or higher. Moreover, 1102 participants (77.3%) met the score criteria for both subscales for burnout. The burnout rate was higher among women (793/923, 85.9%) than men (309/502, 61.6%), and higher among the 20–29 years age group (480/581, 82.6%) than in the 30–39 years age group (440/565, 77.9%). Regarding employment duration, the burnout rate was high in the one to five years group (544/686, 79.3%) and in the six to ten years group (223/279, 79.9%). Regarding occupation, nurses had the highest burnout rate (633/712, 88.9%), followed by physicians (128/167, 76.6%), hospital administrative staff, epidemiologists, and civic servants (80/114, 70.2%), paramedics (184/297, 62.0%), nurse aids, transport staff, cleaning staff, radiologic technologists, and clinical pathologists (77/135, 57.0%).

An supplementary table about the burnout rate according to the job position and clinical career of physicians shows that among the doctors, the burnout rate was the highest among residents (71/82, 86.6%) [see Table S1 in Supplementary Tables file], and another table about burnout rate according to the job position and clinical career of nurses shows that among the nurses, the burnout rate was the highest among charge nurses (28/29, 96.6%), followed by staff nurses (556/612, 89.5%) [see Table S2 in Supplementary Tables file].

### Burnout according to COVID-19-related work and work experience

Table 3 shows the burnout rate of healthcare workers according to COVID-19-related work. Among the total respondents, 1066 (74.8%) had direct contact with COVID-19 patients (e.g., nurse, doctor, transport staff, testing staff, and cleaning staff). Of these 1066 participants, 855 (80.2%) experienced burnout, which is a higher burnout rate than healthcare workers working without direct contact with COVID-19 patients (247/359, 68.8%). According to an average contact time with COVID-19 patients per day, the burnout rate was the highest among workers who had an average two to six hours of exposure to patients (311/349, 89.1%), and according to days of care for COVID-19 patients, the burnout rate was the highest in 90 days or longer group (368/440, 83.6%). According to work location, healthcare workers who worked in the COVID-19 ward (399/471, 84.7%) and worked in the emergency room (167/201, 83.1%) had a higher burnout rate than those who did not. Those who worked in the ambulance (156/228, 68.4%) had a lower burnout rate than those who did not. According to the work experience with difficult COVID-19 patients, the burnout rates of those with experience in caring for critically ill and mentally ill patients were 83.6% (398/476) and 83.7% (484/578), higher than those without experience. In addition, the burnout rates of those with experience wearing level D PPE and powered air-purifying respirators (PAPR) were 82.2% (525/639) and 84.5% (523/619), higher than those without experience.

**Table 3:** Burnout rate according to COVID-19-related work experience.

Variable	Total (N = 1425)	Burnout (N = 1102)	No Burnout (N = 323)	*P*-value
Worked in direct contact with COVID-19 patients				*P* < 0.001
Yes	1066	855 (80.2%)	211 (19.8%)	
No	359	247 (68.8%)	112 (31.2%)	
Currently working in direct contact with COVID-19 patients				0.016
Yes	556	449 (80.8%)	107 (19.2%)	
No	869	653 (75.1%)	216 (24.9%)	
Average contact time with COVID-19 patients per day				*P* < 0.001
Not at all	369	253 (68.6%)	116 (61.4%)	
< 30 min	269	205 (76.2%)	64 (23.8%)	
30 min–2 h	349	261 (74.8%)	88 (25.2%)	
2–6 h	349	311 (89.1%)	38 (10.9%)	
> 6 h	89	72 (80.9%)	17 (19.1%)	
Number of COVID-19 patients cared for				*P* < 0.001
0	366	252 (68.9%)	114 (31.1%)	
1–10	409	324 (79.2%)	85 (20.8%)	
11–20	145	119 (82.1%)	26 (17.9%)	
> 21	505	407 (80.6%)	98 (19.4%)	
Days of care for COVID-19 patients				*P* < 0.001
0	373	257 (68.9%)	116 (31.1%)	
1–29 days	403	309 (76.7%)	94 (23.3%)	
30–59 days	115	92 (80.0%)	23 (20.0%)	
60–89 days	94	76 (80.9%)	18 (19.1%)	
≥ 90 days	440	368 (83.6%)	72 (16.4%)	
Work location				
COVID-19 ward				*P* < 0.001
Yes	471	399 (84.7%)	72 (15.3%)	
No	954	703 (73.7%)	251 (26.3%)	
Emergency room				0.036
Yes	201	167 (83.1%)	34 (16.9%)	
No	1224	935 (76.4%)	289 (23.6%)	
COVID-19 intensive care unit				0.238
Yes	312	249 (79.8%)	63 (20.2%)	
No	1113	853 (76.6%)	260 (23.4%)	
COVID-19 screening center				0.544
Yes	163	123 (75.5%)	40 (24.5%)	
No	1262	979 (77.6%)	283 (22.4%)	
COVID-19 community care center				0.398
Yes	35	25 (71.4%)	10 (28.6%)	
No	1390	1077 (77.5%)	313 (22.5%)	
Ambulance				*P* < 0.001
Yes	228	156 (68.4%)	72 (31.6%)	
No	1197	946 (79.0%)	251 (21.0%)	
Work experience with difficult COVID-19 patients				
Critically ill				*P* < 0.001
Yes	476	398 (83.6%)	78 (16.4%)	
No	949	704 (74.2%)	245 (25.8%)	
Dementia, delirium, other mental illness				*P* < 0.001
Yes	578	484 (83.7%)	94 (16.3%)	
No	847	618 (73.0%)	229 (27.0%)	
Experience with wearing PPE				
Level D PPE*				*P* < 0.001
Yes	639	525 (82.2%)	114 (17.8%)	
No	786	577 (73.4%)	209 (26.6%)	
PAPR**				*P* < 0.001
Yes	619	523 (84.5%)	96 (15.5%)	
No	806	579 (71.8%)	227 (28.2%)	

*PPE personal protection equipment.

**PAPR powered air-purifying respirator.

### Burnout according to physical and psychological symptoms

Table 4 shows the burnout rate of healthcare workers according to physical, chronic fatigue, depression, and anxiety symptoms; mental disorder diagnosis, PTSD symptoms; GARS score; and positive resources before and after the COVID-19 outbreak. A total of 103 out of 110 participants (93.6%) who had physical symptoms (PHQ-15 ≥ 10) since before the outbreak, experienced burnout, and 407 out of 426 participants (95.5%) with physical symptoms (PHQ-15 ≥ 10) after the outbreak, experienced burnout. At any time before or after the onset of COVID-19, the burnout rate among healthcare workers with physical symptoms (PHQ-15 ≥ 10) was higher than those without physical symptoms (PHQ-15 ≥ 10). A total of 791 of 887 participants (89.2%) who had chronic fatigue symptoms (FSS ≥ 3.22) since before the outbreak experienced burnout, and 964 of 1089 participants (88.5%) with chronic fatigue symptoms (FSS ≥ 3.22) after the outbreak experienced burnout. Chronic fatigue symptoms, like physical symptoms, had a higher rate of burnout in those who had symptoms at any time before or after the onset of COVID-19. The burnout rates among those with depression symptoms (PHQ-9 ≥ 10) since before and after the outbreak were 94.4% (51/54) and 96.4% (268/278), respectively, and the burnout rate was 100% among those who had anxiety symptoms (GAD-7 ≥ 10) since before (n = 28) and after (n = 43) the outbreak. In total, burnout was reported by 39 of 41 (95.2%) and 18 of 20 (90.0%) participants diagnosed with a mental disorder before and after the outbreak, respectively. Regarding PTSD symptoms within the month before the survey period, 158 of 171 (92.4%) participants who experienced moderate PTSD symptoms (PC-PTSD-5 = 2), and 282 of 299 (94.3%) participants who experienced severe PTSD symptoms (PC-PTSD-5 ≥ 3), experienced burnout. The burnout rate among those with insomnia was 94.9% (277/292), while it was 72.8% (825/1,133) in those without insomnia.

**Table 4:** Burnout rate according to the presence of physical and mental symptoms before and after the COVID-19 pandemic.

Variable	Total (N = 1425)	Burnout (N = 1102)	No Burnout (N = 323)	*P*-value
Physical symptoms (PHQ-15 ≥ 10)				
Before the COVID-19 pandemic				*P* < 0.001
Yes	110	103 (93.6%)	7 (6.3%)	
No	1315	999 (76.0%)	316 (24.0%)	
After the COVID-19 pandemic				*P* < 0.001
Yes	426	407 (95.5%)	19 (4.5%)	
No	999	695 (69.6%)	304 (30.4%)	
Chronic fatigue symptoms (FSS ≥ 3.22)				
Before the COVID-19 pandemic				*P* < 0.001
Yes	887	791 (89.2%)	96 (10.8%)	
No	538	311 (57.8%)	227 (42.2%)	
After the COVID-19 pandemic				*P* < 0.001
Yes	1089	964 (88.5%)	125 (11.5%)	
No	336	138 (41.1%)	198 (58.9%)	
Depression symptoms (PHQ-9 ≥ 10)				
Before the COVID-19 pandemic				0.004
Yes	54	51 (94.4%)	3 (5.6%)	
No	1371	1051 (76.7%)	320 (23.3%)	
After the COVID-19 pandemic				*P* < 0.001
Yes	278	268 (96.4%)	10 (3.6%)	
No	1147	834 (72.7%)	282 (27.3%)	
Anxiety symptoms (GAD-7 ≥ 10)				
Before the COVID-19 pandemic				0.004
Yes	28	28 (100%)	0 (0.0%)	
No	1397	1074 (76.9%)	323 (23.1%)	
After the COVID-19 pandemic				*P* < 0.001
Yes	43	43 (100%)	0 (0.0%)	
No	1382	1059 (76.6%)	323 (23.4%)	
Diagnosis of mental disorders before the COVID-19 pandemic				0.006
Yes	41	39 (95.2%)	2 (4.8%)	
No	1384	1063 (76.8%)	321 (23.2%)	
Diagnosis of mental disorders after the COVID-19 pandemic				0.2793
Yes	20	18 (90.0%)	2 (10.0%)	
No	1405	1084 (77.2%)	321 (22.8%)	
Symptoms of post-traumatic stress symptoms in the past month (PC-PTSD-5 ≥ 2)				*P* < 0.001
Normal	955	662 (69.3%)	293 (30.7%)	
Mild-moderate	171	158 (92.4%)	13 (7.6%)	
Severe	299	282 (94.3%)	17 (5.7%)	
Insomnia				*P* < 0.001
Yes	292	277 (94.9%)	15 (5.1%)	
No	1133	825 (72.8%)	308 (27.2%)	

### Burnout according to stress and positive resources

Table 5 shows the GARS and POREST scores according to burnout. The mean GARS score was 3.2 among 1102 participants with burnout, 1.7 points higher than that among 323 participants without burnout, indicating that those experiencing burnout had more significant perceived stress. In particular, the stress score for job in the burnout group was 4.7, the highest among all GARS subscale scores. The POREST score was 78.5 in the burnout group and 89.9 in the non-burnout group. The burnout group scored higher than the non-burnout group in all items of the POREST.

**Table 5:** The mean (SD) GARS scale and POREST scores according to burnout.

Variable	Total (N = 1425) Mean (SD)	Burnout (N = 1102) Mean (SD)	No Burnout (N = 323) Mean (SD)	*P*-value
GARS scale				
Average	2.9 (1.6)	3.2 (1.5)	1.7 (1.0)	*P* < 0.001
Job, School	4.2 (2.1)	4.7 (1.9)	2.5 (1.6)	*P* < 0.001
Family interpersonal	3.1 (2.1)	3.5 (2.1)	1.9 (1.5)	*P* < 0.001
Changes in relationships	2.7 (2.2)	3.0 (2.3)	1.6 (1.6)	*P* < 0.001
Sickness, injury	3.1 (2.2)	3.5 (2.2)	1.8 (1.7)	*P* < 0.001
Financial	2.9 (2.2)	3.2 (2.2)	1.9 (1.8)	*P* < 0.001
Unusual events	2.3 (2.0)	2.5 (2.1)	1.3 (1.4)	*P* < 0.001
Change routine	1.6 (1.7)	1.8 (1.8)	0.8 (1.0)	*P* < 0.001
Overall global	3.0 (2.1)	3.5 (2.1)	1.4 (1.3)	*P* < 0.001
POREST scores				
Optimism	24.7 (4.5)	23.7 (4.2)	28.1 (3.6)	*P* < 0.001
Purpose and hope	20.7 (3.9)	20.0 (3.9)	22.7 (3.5)	*P* < 0.001
Self-control	16.9 (3.2)	16.4 (3.1)	18.9 (2.7)	*P* < 0.001
Social resource support	11.5 (2.1)	11.3 (2.2)	12.4 (1.8)	*P* < 0.001
Care	7.3 (1.5)	7.1 (1.5)	7.8 (1.4)	*P* < 0.001
Total	81.1 (12.7)	78.5 (12.1)	89.9 (10.6)	*P* < 0.001

*GARS* global assessment of recent stress scale, *POREST* positive resources test.

### Correlates of burnout

Table 6 shows the correlates of burnout using univariate and multivariable logistic regression. In this study, gender, age, physical symptoms and chronic fatigue symptoms after the COVID-19 outbreak, PTSD symptoms, and GARS score were related to burnout. In multiple regression model adjusted for other confounders, the odds for burnout were 2.05 times higher for women (95% confidence interval [CI] = 1.46–2.86; *p* < 0.001) than men and 1.45 times higher in younger groups than older ones (95% CI = 1.22–1.72; *p* < 0.001). The odds for burnout were 2.03 times higher (95% CI = 1.14–3.60; *p* = 0.016) among those who developed physical symptoms (PHQ-15 ≥ 10) after the COVID-19 outbreak and 3.94 times higher (95% CI 2.80–5.56; *p* < 0.001) among those with chronic fatigue symptoms (FSS ≥ 3.22; *p* < 0.001). The odds for burnout were 1.47 times higher (95% CI 1.08–2.01; *p* = 0.014) in the groups that experienced moderate or severe PTSD symptoms (PC-PTSD-5 ≥ 2) than in the non-PTSD group. The odds for burnout increased by 1.71 times (95% CI 1.46–2.01; *p* < 0.001) with a one-point increase in the GARS score. Regarding the factors of positive resources, optimism and caring were found to significantly reduce the risk for burnout, with a one-point increase in both optimism and caring decreasing the odds for burnout by 0.84 times (95% CI 0.80–0.88; *p* < 0.001) and 0.86 times (95% CI 0.77–0.99; *p* = 0.030), respectively. The AUC value of the multivariable logistic regression model was acceptable (0.893).

**Table 6:** Logistic regression to identify the correlates of burnout.

Variable	Univariate logistic regression analysis		Multivariable logistic regression analysis	
	OR (95% CI)	*P*-value	OR (95% CI)	*P*-value
Women (compared to men)	3.81 (2.94, 4.93)	*P* < 0.001	2.05 (1.46, 2.86)	*P* < 0.001
Age*	1.48 (1.31, 1.69)	*P* < 0.001	1.45 (1.22, 1.72)	*P* < 0.001
Physical symptoms** after COVID-19 pandemic	9.37 (5.80, 15.13)	*P* < 0.001	2.03 (1.14, 3.60)	0.016
Chronic fatigue symptoms*** after COVID-19 pandemic	11.07 (8.31, 17.73)	*P* < 0.001	3.94 (2.80, 5.56)	*P* < 0.001
Post-traumatic stress symptoms****	3.09 (2.40, 3.98)	*P* < 0.001	1.47 (1.08, 2.01)	0.014
GARS Scale (for every 1-point increase)	2.79 (2.42, 3.21)	*P* < 0.001	1.71 (1.46, 2.01)	*P* < 0.001
Optimism score of POREST (for every 1-point increase)	0.75 (0.72, 0.78)	*P* < 0.001	0.84 (0.80, 0.88)	*P* < 0.001
Caring score of POREST (for every 1-point increase)	0.74 (0.67, 0.81)	*P* < 0.001	0.87 (0.77, 0.99)	0.030

*CI* confidence interval, *GARS* global assessment of recent stress scale, *POREST* positive resources test.

*60 years and older, 50–59 years, 40–49 years, 30–39 years, 20–29 years, as the age group decreases from the older age group to the lower age group.

**Physical symptoms mean a score of 10 or higher on the Patient Health Questionnaire-15.

***Chronic fatigue symptoms mean a score of 3.22 or higher on the Fatigue Severity Scale.

****Post-traumatic stress symptoms mean a score of 2 or higher on the Primary Care Post-Traumatic Stress Disorder-5 scale.

### Physical (PHQ-15 ≥ 10) and chronic fatigue (FSS ≥ 3.22) symptoms after the COVID-19 outbreak according to patient care work

Tables 7 and 8 show the predictors of physical symptoms (PHQ-15 ≥ 10) and chronic fatigue symptoms (FSS ≥ 3.22) after the COVID-19 outbreak according to patient care work using univariate and multivariable logistic regression. The odds for physical symptoms (PHQ-15 ≥ 10) and chronic fatigue symptoms (FSS ≥ 3.22) after the COVID-19 outbreak were 2.04 times (95% CI = 1.52–2.78; *p* < 0.001) and 2.14 times (95% CI = 1.52–3.02; *p* < 0.001) higher among those who worked in medical institutions as frontline workers (COVID-19 ward, emergency room and COVID-19 intensive care unit) in multivariable logistic regression analysis.

**Table 7:** Univariate logistic analysis of physical (PHQ-15 ≥ 10) and chronic fatigue (FSS ≥ 3.22) symptoms after the COVID-19 outbreak according to patient care work.

Variable	Univariate logistic analysis			
	Physical symptoms		Chronic fatigue symptoms	
	OR (95% CI)	*P*-value	OR (95% CI)	*P*-value
Work location				
Medical institutions (COVID-19 ward, emergency room, COVID-19 intensive care unit)	2.46 (1.93, 3.14)	*P* < 0.001	2.22 (1.73, 2.85)	*P* < 0.001
Non-medical institutions (residential treatment center, ambulance, others)	0.46 (0.34, 0.62)	*P* < 0.001	0.67 (0.51, 0.89)	0.005
Work experience with difficult COVID-19 patient				
Critically ill	1.81 (1.43, 2.30)	*P* < 0.001	1.62 (1.23, 2.13)	*P* < 0.001
Dementia, delirium, other mental illness	2.16 (1.72, 2.73)	*P* < 0.001	1.91 (1.47, 2.49)	*P* < 0.001
Number of COVID-19 patients cared for*	1.24 (1.13, 1.36)	*P* < 0.001	1.21 (1.09, 1.34)	*P* < 0.001
Days of care for COVID-19 patients**	1.26 (1.17, 1.35)	*P* < 0.001	1.22 (1.13, 1.32)	*P* < 0.001
Currently working in direct contact with COVID-19 patient	1.81 (1.44, 2.28)	*P* < 0.001	1.61 (1.24, 2.09)	*P* < 0.001
Average contact time with COVID-19 patients per day***	1.37 (1.25, 1.51)	*P* < 0.001	1.29 (1.17, 1.43)	*P* < 0.001
Experience with wearing Level D PPE	1.63 (1.29, 2.04)	*P* < 0.001	1.56 (1.21, 2.01)	*P* < 0.001
Experience with wearing PAPR	1.77 (1.41, 2.22)	*P* < 0.001	1.90 (1.47, 2.46)	*P* < 0.001

*CI* confidence interval, *PPE* personal protection equipment, *PAPR* powered air-purifying respirator.

*0, 1–10 patients, 11–20 patients, more than 20 patients, as the number of patients to care increases from a small group to a large group.

**0, 1–29 days, 30–59 days, 60–89 days, more than 90 days, as the number of days of care for COVID-19 patients increases from a small group to a large group.

***No, less than 30 min, 30 min–2 h, 2–6 h, more than 6 h, as the contact time with COVID-19 patients per day increases from a small group to a large group.

**Table 8:** Multivariable logistic regression analysis of physical (PHQ-15 ≥ 10) and chronic fatigue (FSS ≥ 3.22) symptoms after the COVID-19 outbreak according to patient care work.

Variable	Multivariable logistic analysis			
	Physical symptoms		Chronic fatigue symptoms	
	OR (95% CI)	*P*-value	OR (95% CI)	*P*-value
Work location				
Medical institutions (COVID-19 ward, emergency room, COVID-19 intensive care unit)	2.04 (1.52, 2.78)	*P* < 0.001	2.14 (1.52, 3.02)	*P* < 0.001
Number of COVID-19 patients cared for*	0.81 (0.67, 0.94)	*P* = 0.026	0.82 (0.68, 0.99)	*P* = 0.034
Days of care for COVID-19 patients**	1.21 (1.06, 1.38)	*P* = 0.004	1.18 (1.02, 1.37)	*P* < 0.001
Average contact time with COVID-19 patients per day***	1.34 (1.17, 1.54)	*P* < 0.001		

*CI* confidence interval.

*0, 1–10 patients, 11–20 patients, more than 20 patients, as the number of patients to care increases from a small group to a large group.

**0, 1–29 days, 30–59 days, 60–89 days, more than 90 days, as the number of days of care for COVID-19 patients increases from a small group to a large group.

***No, less than 30 min, 30 min–2 h, 2–6 h, more than 6 h, as the contact time with COVID-19 patients per day increases from a small group to a large group.

### Exhaustion score and disengagement score according to multiple variables

We analyzed correlation between scores for each of the two domains of burnout, exhaustion and disengagement, and clinical variables including demographic characteristics. Tables 9 and 10 show the strength of adjusted associations from linear regression analysis between the covariates and the score of each domains of burnout. The predictors explained 57.6% of exhaustion score and 46.9% of disengagement score. Chronic fatigue symptoms after the outbreak of COVID-19 had the strongest relationship with both exhaustion (standardized β = 1.96; *p* < 0.001) and disengagement score (standardized β = 1.89; *p* < 0.001). Consistent with the results in previous paragraphs, in addition to chronic fatigue symptoms after COVID-19, women, younger age, and GARS score were positively correlated with both exhaustion and disengagement score.

**Table 9:** Multiple linear regression analyses predicting exhaustion score.

Variable	Exhaustion multiple regression	
	Standardized β (95% CI)	*P*-value
Women (compared to men)	0.70 (0.38, 1.02)	*P* < 0.001
Age*	0.45 (0.29, 0.60)	*P* < 0.001
Physical symptoms** after COVID-19 pandemic	0.51 (0.14, 0.88)	0.007
Chronic fatigue symptoms*** after COVID-19 pandemic	1.96 (1.59, 2.33)	*P* < 0.001
Post-traumatic stress symptoms****	0.28 (0.07, 0.48)	0.009
Depression symptoms***** before COVID-10 pandemic	− 1.14 (− 1.91, − 0.37)	0.004
Depression symptoms***** after COVID-10 pandemic	0.88 (0.44, 1.33)	*P* < 0.001
Presence of insomnia	0.54 (0.15, 0.93)	0.007
GARS Scale (for every 1-point increase)	0.58 (0.47, 0.69)	*P* < 0.001
Work at COVID-19 ward (compared to other location)	0.43 (0.11, 0.75)	0.009
Optimism score of POREST (for every 1-point increase)	− 0.23 (− 0.28, − 0.18)	*P* < 0.001
Self-control score of POREST (for every 1-point increase)	− 0.13 (− 0.19, − 0.07)	*P* < 0.001
Social resource support score of POREST (for every 1-point increase)	0.11 (0.02, 0.19)	0.018
Adjusted R square	0.576	*P* < 0.001

*CI* confidence interval, *GARS* global assessment of recent stress scale, *POREST* positive resources test.

*60 years and older, 50–59 years, 40–49 years, 30–39 years, 20–29 years, as the age group decreases from the older age group to the lower age group.

**Physical symptoms mean a score of 10 or higher on the Patient Health Questionnaire-15.

***Chronic fatigue symptoms mean a score of 3.22 or higher on the Fatigue Severity Scale.

****Post-traumatic stress symptoms mean a score of 2 or higher on the Primary Care Post-Traumatic Stress Disorder-5 scale.

**Table 10:** Multiple linear regression analyses predicting disengagement score.

Variable	Disengagement multiple regression	
	Standardized β (95% CI)	P-value
Women (compared to men)	0.64 (0.32, 0.96)	*P* < 0.001
Age*	0.49 (0.30, 0.68)	*P* < 0.001
Length of current employment		
1–5 years	0.46 (0.00, 0.92)	0.052
6–10 years	0.65 (0.12, 1.19)	0.017
> 10 years	0.81 (0.24, 1.34)	0.006
Chronic fatigue symptoms** before COVID-19 pandemic	− 0.47 (− 0.89, − 0.05)	0.027
Chronic fatigue symptoms** after COVID-19 pandemic	1.89 (1.39, 2.38)	*P* < 0.001
Depression symptoms*** after COVID-10 pandemic	0.90 (0.49, 1.32)	*P* < 0.001
Anxiety symptoms**** before COVID-19 pandemic	− 1.68 (− 2.75, − 0.61)	0.002
GARS Scale (for every 1-point increase)	0.41 (0.29, 0.52)	*P* < 0.001
Presence of mental disorders before the COVID-19 pandemic	1.10 (0.22, 1.98)	0.015
Optimism score of POREST (for every 1-point increase)	− 0.19 (− 0.24, − 0.13)	*P* < 0.001
Purpose and hope score of POREST (for every 1-point increase)	− 0.20 (− 0.26, − 0.15)	*P* < 0.001
Social resource support score of POREST (for every 1-point increase)	0.23 (0.14, 0.33)	*P* < 0.001
Caring score of POREST (for every 1-point increase)	− 0.28 (− 0.40, − 0.17)	*P* < 0.001
Adjusted R square	0.469	*P* < 0.001

*CI* confidence interval, *GARS* global assessment of recent stress scale, *POREST* positive resources test.

*60 years and older, 50–59 years, 40–49 years, 30–39 years, 20–29 years, as the age group decreases from the older age group to the lower age group.

**Physical symptoms mean a score of 10 or higher on the Patient Health Questionnaire-15.

***Depression symptoms mean a score of 10 or higher on the Patient Health Questionnaire-9.

****Anxiety symptoms mean a score of 10 or higher on the General Anxiety Disorder-7.

## Discussion

In our study, of 1425 healthcare workers, 1204 reported feeling exhausted (84.5%), and 1298 (91.1%) were disengaged; 1102 (77.3%) met both the criteria and were thus deemed to have burnout. These numbers are higher than the exhaustion (65.5%) and disengagement (79.5%) rates reported by 171 healthcare workers surveyed during the Middle East respiratory syndrome (MERS) epidemic in Korea from 2015 to 2016^25^. In addition, the burnout rate in our study was higher than in previous COVID-19-related studies of healthcare workers conducted in Singapore, India, the United Kingdom, and Poland^16,17,46,47^. However, the burnout assessments for healthcare workers in those countries were performed in March–June 2020, approximately six months before our study. The duration of the MERS epidemic in Korea was less than three months (May 2015 to July 28, 2015). Taken together, these results suggest that healthcare worker burnout has been exacerbated by the prolonged duration of the COVID-19 pandemic. A recently published systematic review and meta-analysis on the psychological distress of healthcare workers treating COVID-19 patients in Asia also speculated that the high burnout rate among healthcare workers during the COVID-19 pandemic, compared to that during the SARS and MERS outbreaks, is due to the prolonged pandemic, as it has persisted for more than a year now^48^. In addition, a Canadian study on burnout among hospital healthcare workers, published in October 2021, reported that the burnout rate among healthcare workers surveyed in the spring of 2021 exceeded 60%, an increase from approximately 30–40% in the spring of 2020, highlighting the urgency of assessing organizational interventions and current systems, and discovering solutions to reduce burnout among healthcare workers^49^.

The burnout rate was higher among women than men in our study. Nurses were at greater risk of exposure to depression, anxiety, and stress because they were primarily involved in the direct care of COVID-19 patients compared to participants in other jobs. This could be attributable to the high-risk of burnout and since most nurses are women, it could explain the high burnout rate among women^50,51^. In addition, stress-related disorders, such as depression and anxiety disorder, are approximately two-fold higher among women than in men^52,53^, suggesting that women may be more vulnerable to depression, anxiety, and stress than men even in a similar environment, which may elevate their risk for burnout^54^.

The burnout rate increased with decreasing age in our study. A Chinese survey on healthcare workers treating COVID-19 patients also reported that the burnout rate was higher in the < 30-years age group than that in the 30–39-years and ≥ 40-years age groups^55^. A Turkish study of nurses during the COVID-19 pandemic also showed that the burnout rate increased with decreasing age^56^. The most significant reason for this result may be that younger nurses are less experienced, and the unfamiliarity of their tasks increases their stress and burnout. Another reason may be that younger individuals tend to be more involved in leisure activities and private social gatherings than their older counterparts, and their burnout may naturally be aggravated due to the restrictions imposed on these activities due to the COVID-19 pandemic^57^. In our study, 581 of 1425 participants (40.8%) were aged 20–29 years, which was the youngest age group of our study population, and 71 of them were physicians or nurses with a clinical career of less than one year, 55 of whom (77.5%) were found to experience burnout. Accordingly, in order to prevent turnover and burnout of new healthcare workers during a pandemic such as COVID-19, it is considered important to provide more education and training to them than trained healthcare workers, and periodically listen to their concerns to improve the work environment.

PTSD manifests as an extreme psychological response to a severe event wherein the individual feels traumatized by continuously reliving the experience. A Norwegian study on PTSD among 1773 healthcare workers during the COVID-19 pandemic reported that PTSD was significantly correlated with burnout^58^. A COVID-19-related study of 2579 healthcare workers in the United States also reported significant correlations among PTSD, burnout, difficulty with work, and interpersonal relationships^59^. A systematic review of 24 studies on PTSD among healthcare workers during SARS, MERS, and COVID-19 outbreaks showed that the risk for PTSD was higher among the frontline staff, those who work at wards with increased exposure to high-risk patients, and those with prolonged contact with patients^60^. The relationship between PTSD symptoms and burnout has been reported in several studies. Several studies have investigated the effect of PTSD on burnout^61,62^ and several studies have investigated the role of burnout in the development of PTSD^63,64^. As mentioned earlier, exposure to traumatic and stressful events can lead to the development of both PTSD symptoms and burnout. Therefore, PTSD symptoms and burnout are closely related but it may be difficult to define a causal relationship. Considering these results, it is necessary to develop measures to reduce exposure to infection risk to reduce both PTSD symptoms and burnout among healthcare workers during the COVID-19 pandemic and enforce regulations on the appropriate duration of contact with patients per healthcare worker.

The presence of Physical (PHQ-15 ≥ 10) and chronic fatigue symptoms (FSS ≥ 3.22) after the outbreak of COVID-19 were statistically significant associations of burnout in our study. Although we evaluated healthcare workers’ physical and chronic fatigue symptoms both since before and after the COVID-19 outbreak, but only the presence of physical and chronic fatigue symptoms after the COVID-19 outbreak are significant positive associations of burnout in a multivariable logistic regression analysis. To identify factors related to physical (PHQ-15 ≥ 10) and chronic fatigue symptoms (FSS ≥ 3.22) after the outbreak of COVID-19, we analyzed significant variables according to the COVID-19 patient care work in Tables 7 and 8. The risk for both symptoms was higher among those working in medical institutions such as the emergency department, intensive care unit (ICU), and COVID-19 ward––areas at high-risk of exposure to COVID-19 patients, with an increased number of days of care work, and increased daily average contact time with COVID-19 patients. Multivariable regression analysis showed that the greater the number of COVID-19 patients to be cared for, the lower the risk of physical symptoms (PHQ-15 ≥ 10) and chronic fatigue symptoms (FSS ≥ 3.22), but there is a limit to interpreting the number of COVID-19 patients to be cared for as proportional to the workload since we surveyed the cumulative number of patients to be cared for up to the time of the survey, not the number of patients to be cared for per day. In addition, when caring for critically ill or mentally ill patients, although the number of patients may be small, the workload may be higher because the severity of illness is high. Experiences with especially difficult patients, such as critically or mentally ill patients, and experiences with level D PPE or PAPR can also be risk predictors associated with physical and chronic fatigue symptoms. An Indian study conducted in December 2020 on the physiological effects of the use of N95 masks and PPE reported that among 75 healthcare workers who vigorously worked in a COVID-19 ICU while wearing an N95 mask and PPE for an average of 3.1 h a day, 90.1%, 70.7%, and 60% suffered from headache, fatigue, and dyspnea, respectively^65^. In addition, a Saudi Arabian survey of 1060 healthcare workers who worked at a hospital during the COVID-19 pandemic reported that the risk of headache increased with the increasing duration of PPE use in people with and without pre-existing headaches. Moreover, the use of PPE also provoked nausea, vomiting, sensitivity to light, sound, and motion, and throat discomfort^66^. In a study on burnout among healthcare workers who worked in a high-risk region in China during the COVID-19 pandemic, physical symptoms and acute stress were significantly correlated with emotional exhaustion and disengagement (cynicism), indicating burnout^67^, and a study in 2001 on the relationship between workload and burnout reported that excessive workload leads to emotional exhaustion, which in turn leads to disengagement (cynicism), which can trigger physical symptoms^68^. These results show that excessive workload can worsen physical symptoms, chronic fatigue symptoms, stress, and burnout, highlighting the urgent need to improve the current working environment. This aligns with the need for improvement of work environment, which is perceived by the participants as most important, as shown in an supplementary table [see Table S3 in Supplementary Tables file]. Thus, measures to lower excessive workload need to be implemented to reduce physical symptoms and chronic fatigue symptoms and ultimately to prevent burnout in healthcare workers treating COVID-19 patients; particularly, standard limits for patient contact and number of days of COVID-19 work need to be established for healthcare workers who work in direct contact with COVID-19 patients.

In this study, the optimism and caring score components of the POREST were identified as significant negative associations with burnout. This means that individuals who were more emotionally positive and had a greater tendency to try to help others had a lower degree of job burnout. The POREST measure was developed in Korea in 2018; although as yet there are few related data, a study on risk factors for burnout conducted in Korea during the MERS epidemic found that the lower the purpose and hope scores of the POREST, the higher the risk of burnout^25^. In another study conducted among 217 clinical nurses at a public hospital in Korea, the relationship between all positive resources of the POREST and burnout showed a significant negative correlation^69^. Therefore, to prevent job burnout of healthcare workers, it is also necessary to evaluate positive resources and develop programs that can increase positive resources.

Currently, countries worldwide are actively vaccinating citizens to eradicate the COVID-19 pandemic and are striving to develop effective therapeutics. Despite such endeavors, the COVID-19 pandemic persists, and healthcare workers responding to this threat are experiencing severe burnout. The WHO Regional Office of Europe published a report on the policies supporting healthcare workers treating COVID-19 patients in several countries in Europe in 2020. The report shows that Malta and Poland had already enforced policies to manage mental health, offered a financial reward, parenting support, and vacation compensation for healthcare workers treating COVID-19 patients since the initial days of the COVID-19 outbreak^70^. Korea had been preparing for the emerging infectious disease pandemic since the outbreak of MERS in 2015, but when the explosive COVID-19 outbreak at the epicenter occurred, there was a shortage of healthcare workers and medical facilities^23^. This shortage has resulted in work overload and burnout for healthcare workers. We need to accurately assess the state of burnout among healthcare workers involved in COVID-19 care and implement measures to reduce their burnout.

This study has the following limitations. First, the questionnaire was administered from December 1, 2020, to January 29, 2021, a period during the third wave, primarily throughout the Seoul metropolitan area with more than 1000 newly diagnosed cases every day. Despite that, only 556 out of 1425 (39%) healthcare workers were involved in COVID-19 care during the survey time. Healthcare workers actively overloaded with COVID-19-related care may not have participated in the survey, which is a limitation of a large-scale questionnaire survey wherein the respondents cannot be selected. Second, the burnout prevalence among Korean healthcare workers before the COVID-19 pandemic could not be assessed, and some questions from a past point in time (before COVID-19) were based on retrospective recalls of the past, there may be limitations. However, even if there is a memory bias for retrospective recalls, it is necessary to consider the physical and psychological symptoms before the outbreak of COVID-19 to more accurately identify risk factors for burnout due to the COVID-19 outbreak. Third, there is a confounding variable that influences both the independent and dependent variables. We used multivariable logistic regression analysis in this study to attempt to adjust as much as possible for potential confounding variables such as gender (e.g., nursing is a female dominated profession), but there may be limitations in interpreting the meaning of the results. In addition, some overlapping items in the self-questionnaire evaluating burnout, chronic fatigue symptoms, physical symptoms, depression symptoms and stress symptoms can increase their correlations with each other, and some variables with overlapping items for burnout can be overestimated as risk factors for burnout. In this study, it was not possible to ascertain causal relationship between burnout and the analyzed confounding variables (e.g., physical symptoms, chronic fatigue symptoms, depression symptoms and PTSD symptoms), and additional research will be needed. Finally, we did not assess the workload of healthcare workers who did not have direct contact with COVID-19 patients, such as COVID-19 epidemiologists and nursing civil servants at public health centers, since we only assessed workers involved with direct care of COVID-19 patients. Future research is needed to investigate healthcare workers who do not provide direct COVID-19 care but who experience burnout due to work overload.

## Conclusion

This study aimed to assess burnout among healthcare workers due to the COVID-19 pandemic and identify burnout risk factors to develop measures to lower its prevalence in this population. The results confirmed that the risk of burnout was higher among women and younger workers. Further, the risk of burnout was higher among those who developed physical (PHQ-15 ≥ 10) and chronic fatigue (FSS ≥ 3.22 symptoms) after the outbreak of COVID-19, those with PTSD symptoms, and those with a higher GARS score while the risk was lower among those with high scores in the optimism and caring component of the POREST. To reduce these factors that aggravate burnout, periodically interviewing healthcare workers to determine the state of burnout and implementing educational and other programs that lower stress and enhance positivity may be helpful. The most crucial aspect, however, is to ease their workload. Healthcare workers in direct contact with COVID-19 patients are physically more strained due to PPE, which further elevates their risk of burnout. Thus, specific limitations on the number of work hours spent in direct contact with COVID-19 patients must be established, and healthcare workers should be guaranteed sufficient break periods. In addition, policies and systems to prevent burnout, such as securing more healthcare personnel and providing compensation, are urgently needed.

## Supplementary Information

Supplementary Information 1.

Supplementary Information 2.

## Data Availability

The datasets used and/or analyzed during the current study available from the corresponding author on reasonable request.

## Abbreviations

COVID-19: Coronavirus disease 2019
WHO: World Health Organization
MERS: Middle East respiratory syndrome
SARS: Severe acute respiratory syndrome
PTSD: Post-traumatic stress disorder
FSS: Fatigue severity scale
PC-PTSD-5: Primary care post-traumatic stress disorder-5
POREST: Positive resources test
GARS: Global assessment of recent stress scale
PPE: Personal protection equipment
PHQ-15: Patient health questionnaire-15
PHQ-9: Patient health questionnaire-9
GAD-7: Generalized anxiety disorder scale
ICU: Intensive care units
PAPR: Powered air-purifying respirator
