# Supplementary material for: Correlates of burnout among healthcare workers during the COVID-19 pandemic in South Korea
Source: Sci Rep. 2023 Feb 27;13:3360. doi: 10.1038/s41598-023-30372-x (PMC9969371; doi:10.1038/s41598-023-30372-x)
Supplement: Supplementary file 1 — Supplementary Information 1. [file 41598_2023_30372_MOESM1_ESM.docx]

Supplementary Note

Supplementary Note for the English translation of the questionnaire for healthcare workers responding to the COVID-19 outbreak

**※ These are questions about your demographics and work-related information. Please choose the best applicable answer.**

| 1. Gender | ① Man ② Woman | |
| --- | --- | --- |
| 2. Age | ① <20 years ② 20–29 years ③ 30–39 years  ④ 40–49 years ⑤ 50–59 years ⑥ ≥60 years | |
| 3. Living arrangement at the time of COVID-19-related work (can choose multiple options ) | ① Lived with family ② Lived in a group environment such as dorm ③ Lived alone ④ Other | |
| 4. Family members who lived with you during the time of COVID-19-related work  (Can choose multiple options) | ① Spouse ② Father ③ Mother ④ Child ⑤ Other co-habitants ⑥ No co-habitants | |
| 5. Current region of work | ① Seoul metropolitan area ② Gangwon ③ Chungcheong ④ Jeolla ⑤ Gyeongsang ⑥ Jeju | |
| 6. Type of facility | Type of facility: | |
|  | Primary care facility | ① Private health clinic ② Public health center |
|  | Secondary care facility | ③ General hospital ④ National/public medical center ⑤ University hospital |
|  | Tertiary care facility | ⑥ University hospital (Tertiary hospital) |
|  | Special hospital | ⑦ Long-term care hospital ⑧ Psychiatric hospital |
|  | Residential treatment center | ⑨ Residential treatment center |
|  | Public emergency medical service | ⑩ Paramedics |
|  | Other: Please specify | ⑪ ( ) |
| 7. Length of current employment | ① <1 year ② 1–5 years ③ 6–10 years ④ 11–15 years  ⑤ 16–20 years ⑥ 21–25 years ⑦ 26–30 years ⑧ ≥31 years | |
| 8. Occupation/profession | ① Physician (questions #9–12) ② Nurse (questions #13–17)  ③ Nurse aid ④ Transport staff ⑤ Cleaning staff ⑥ Radiologic technologist ⑦ Hospital administrative staff  ⑧ Epidemiologist ⑨ 119 paramedic ⑩ Civil servant ⑪ Other ( ) | |
| 9. Job position | ① Specialist ② Resident ③ Intern ④ Public health physician ⑤ Military physician | |
| 10. Specialty | ① Emergency medicine ② Infectious diseases ③ Pulmonology  ④ Other internal medicine specialties (including psychiatry) ⑤ Surgery ⑥ Other__________ | |
| 11. Clinical career  (since being licensed) | ① <1 year ② 1–5 years ③ 6–10 years ④ 11–15 years  ⑤ 16–20 years ⑥ 21–25 years ⑦ 26–30 years ⑧ ≥31 years | |
| 12. Career in specialty  (since obtaining specialty certification) | ① <1 year ② 1–5 years ③ 6–10 years ④ 11–15 years  ⑤ 16–20 years ⑥ 21–25 years ⑦ 26–30 years ⑧ ≥31 years | |
| 13. Job position | ① Staff nurse ② Charge nurse ③ Nurse manager (or head nurse)  ④ Other ( ) | |
| 14. Clinical career  (total nursing career) | ① <1 year ② 1–5 years ③ 6–10 years ④ 11–15 years  ⑤ 16–20 years ⑥ 21–25 years ⑦ 26–30 years ⑧ ≥31 years | |
| 15. Work unit | ① Ward ② Outpatient ③ ICU ④ OR  ⑤ ED ⑥ Other ( ) | |
| 16. Length of career at current work unit | ① <1 year ② 1–5 years ③ 6–10 years ④ 11–15 years  ⑤ 16–20 years ⑥ 21–25 years ⑦ 26–30 years ⑧ ≥31 years | |
| 17. Shift work | ① Yes ② No | |

**(Compared to before the COVID-19 pandemic) During the past four weeks, how much have you been bothered by any of the following problems (0-not bothered at all; 1-bothered a little; 2-bothered a lot).**

|  | **Item** | | 0 | 1 | 2 |
| --- | --- | --- | --- | --- | --- |
| 1 | Before COVID-19 | Stomach pain |  |  |  |
|  | During the past four weeks | Stomach pain |  |  |  |
| 2 | Before COVID-19 | Back pain |  |  |  |
|  | During the past four weeks | Back pain |  |  |  |
| 3 | Before COVID-19 | Pain in your arms or legs or other joints |  |  |  |
|  | During the past four weeks | Pain in your arms or legs or other joints |  |  |  |
| 4 | Before COVID-19 | Menstrual cramps or other problems with your periods (women only) |  |  |  |
|  | During the past four weeks | Menstrual cramps or other problems with your periods (women only) |  |  |  |
| 5 | Before COVID-19 | Headaches |  |  |  |
|  | During the past four weeks | Headaches |  |  |  |
| 6 | Before COVID-19 | Chest pain |  |  |  |
|  | During the past four weeks | Chest pain |  |  |  |
| 7 | Before COVID-19 | Dizziness |  |  |  |
|  | During the past four weeks | Dizziness |  |  |  |
| 8 | Before COVID-19 | Fainting spells |  |  |  |
|  | During the past four weeks | Fainting spells |  |  |  |
| 9 | Before COVID-19 | Feeling your heart pound or race |  |  |  |
|  | During the past four weeks | Feeling your heart pound or race |  |  |  |
| 10 | Before COVID-19 | Shortness of breath |  |  |  |
|  | During the past four weeks | Shortness of breath |  |  |  |
| 11 | Before COVID-19 | Pain or problems during sexual intercourse |  |  |  |
|  | During the past four weeks | Pain or problems during sexual intercourse |  |  |  |
| 12 | Before COVID-19 | Constipation, loose bowels, or diarrhea |  |  |  |
|  | During the past four weeks | Constipation, loose bowels, or diarrhea |  |  |  |
| 13 | Before COVID-19 | Nausea, gas, or indigestion |  |  |  |
|  | During the past four weeks | Nausea, gas, or indigestion |  |  |  |
| 14 | Before COVID-19 | Feeling tired, or having low energy |  |  |  |
|  | During the past four weeks | Feeling tired, or having low energy |  |  |  |
| 15 | Before COVID-19 | Trouble sleeping |  |  |  |
|  | During the past four weeks | Trouble sleeping |  |  |  |

**(Compared to before the COVID-19 pandemic) During the past week, choose a number from 1 to 7 that indicates your degree of agreement with the following statements where 1 indicates “strongly disagree” and 7 indicates “strongly agree.”**

|  | **Item** | | **Disagree** **Agree** | | | | | | | | | | | | |
| --- | --- | --- | --- | --- | --- | --- | --- | --- | --- | --- | --- | --- | --- | --- | --- |
|  |  |  | **1** | **2** | | **3** | | **4** | | **5** | | **6** | | **7** | |
| 1 | Before COVID-19 | My motivation is lower when I am fatigued. |  | |  | |  | |  | |  | |  | |  |
|  | During the past four weeks | My motivation is lower when I am fatigued. |  | |  | |  | |  | |  | |  | |  |
| 2 | Before COVID-19 | Exercise brings on fatigue. |  | |  | |  | |  | |  | |  | |  |
|  | During the past four weeks | Exercise brings on fatigue. |  | |  | |  | |  | |  | |  | |  |
| 3 | Before COVID-19 | I am easily fatigued. |  | |  | |  | |  | |  | |  | |  |
|  | During the past four weeks | I am easily fatigued. |  | |  | |  | |  | |  | |  | |  |
| 4 | Before COVID-19 | Fatigue interferes with my physical functioning. |  | |  | |  | |  | |  | |  | |  |
|  | During the past four weeks | Fatigue interferes with my physical functioning. |  | |  | |  | |  | |  | |  | |  |
| 5 | Before COVID-19 | Fatigue causes frequent problems for me. |  | |  | |  | |  | |  | |  | |  |
|  | During the past four weeks | Fatigue causes frequent problems for me. |  | |  | |  | |  | |  | |  | |  |
| 6 | Before COVID-19 | My fatigue prevents sustained physical functioning. |  | |  | |  | |  | |  | |  | |  |
|  | During the past four weeks | My fatigue prevents sustained physical functioning. |  | |  | |  | |  | |  | |  | |  |
| 7 | Before COVID-19 | Fatigue interferes with carrying out certain duties and responsibilities. |  | |  | |  | |  | |  | |  | |  |
|  | During the past four weeks | Fatigue interferes with carrying out certain duties and responsibilities. |  | |  | |  | |  | |  | |  | |  |
| 8 | Before COVID-19 | Fatigue is among my three most disabling symptoms. |  | |  | |  | |  | |  | |  | |  |
|  | During the past four weeks | Fatigue is among my three most disabling symptoms. |  | |  | |  | |  | |  | |  | |  |
| 9 | Before COVID-19 | Fatigue interferes with my work, family, or social life. |  | |  | |  | |  | |  | |  | |  |
|  | During the past four weeks | Fatigue interferes with my work, family, or social life. |  | |  | |  | |  | |  | |  | |  |

**※ This is a questionnaire to look at changes in your mental health after engaging in COVID-19 work.**

**Even if you have not been diagnosed and treated in a hospital, please respond to the items below that you think occurred after you were engaged in work due to COVID-19.**

|  | **Item** | **None** | **Persisted from before COVID-19** | **New or worsening symptoms** |
| --- | --- | --- | --- | --- |
| 1 | Depression, decreased motivation |  |  |  |
| 2 | Anxiety, nervousness |  |  |  |
| 3 | Insomnia |  |  |  |
| 4 | Poor concentration, memory loss |  |  |  |
| 5 | Anger |  |  |  |
| 6 | Drinking problems, alcohol abuse |  |  |  |
| 7 | Exhaustion, burnout |  |  |  |
| 8 | Guilt, self-accusation |  |  |  |
| 9 | Other: Please write your own. ( ) | | | |

**In the past month, how much you have suffered from a stressful event related to COVID-19?**

| **Item** | | **Yes** | **No** |
| --- | --- | --- | --- |
|  |  | **1** | **2** |
| 1 | Had nightmares about the event(s) or thought about the event(s) when you did not want to? |  |  |
| 2 | Tried hard not to think about the event(s) or went out of your way to avoid situations that reminded you of the event(s)? |  |  |
| 3 | Been constantly on guard, watchful, or easily startled? |  |  |
| 4 | Felt numb or detached from people, activities, or your surroundings? |  |  |
| 5 | Felt guilty or unable to stop blaming yourself or others for the events(s) or any problems the event(s) may have caused? |  |  |

**(Compared to before the COVID-19 pandemic) Please respond to the depressive symptoms you have experienced in the past two weeks.**

|  | **Item** | | **Not at all** | **Several days** | **More than half the days** | **Nearly every day** |
| --- | --- | --- | --- | --- | --- | --- |
|  |  |  | **0** | **1** | **2** | **3** |
| 1 | Before COVID-19 | Little interest or pleasure in doing things. |  |  |  |  |
|  | During the past two weeks | Little interest or pleasure in doing things. |  |  |  |  |
| 2 | Before COVID-19 | Feeling down, depressed, or hopeless. |  |  |  |  |
|  | During the past two weeks | Feeling down, depressed, or hopeless. |  |  |  |  |
| 3 | Before COVID-19 | Trouble falling or staying asleep, or sleeping too much. |  |  |  |  |
|  | During the past two weeks | Trouble falling or staying asleep, or sleeping too much. |  |  |  |  |
| 4 | Before COVID-19 | Feeling tired or having little energy. |  |  |  |  |
|  | During the past two weeks | Feeling tired or having little energy. |  |  |  |  |
| 5 | Before COVID-19 | Poor appetite or overeating. |  |  |  |  |
|  | During the past two weeks | Poor appetite or overeating. |  |  |  |  |
| 6 | Before COVID-19 | Feeling bad about yourself, or that you are a failure or have let yourself or your family down. |  |  |  |  |
|  | During the past two weeks | Feeling bad about yourself, or that you are a failure or have let yourself or your family down. |  |  |  |  |
| 7 | Before COVID-19 | Trouble concentrating on things, such as reading the newspaper or watching television. |  |  |  |  |
|  | During the past two weeks | Trouble concentrating on things, such as reading the newspaper or watching television. |  |  |  |  |
| 8 | Before COVID-19 | Moving or speaking so slowly that other people could have noticed, or so fidgety or restless that you have been moving a lot more than usual. |  |  |  |  |
|  | During the past two weeks | Moving or speaking so slowly that other people could have noticed, or so fidgety or restless that you have been moving a lot more than usual. |  |  |  |  |
| 9 | Before COVID-19 | Thoughts that you would be better off dead, or thoughts of hurting yourself in some way. |  |  |  |  |
|  | During the past two weeks | Thoughts that you would be better off dead, or thoughts of hurting yourself in some way. |  |  |  |  |

| **※** If you checked off any problems, how difficult have these problems made it for you to do your work, take care of things at home, or get along with other people? | | | | |
| --- | --- | --- | --- | --- |
| Before  COVID-19 | ① Not difficult at all | ② Somewhat difficult | ③ Very difficult | ④ Extremely difficult |
| During the past  two weeks | ① Not difficult at all | ② Somewhat difficult | ③ Very difficult | ④ Extremely difficult |

**(Compared to before the COVID-19 pandemic) Please respond to any of the symptoms of anxiety you have experienced in the past two weeks.**

|  | **Item** | | **Not at all** | **Several days** | **More than half the days** | **Nearly every day** |
| --- | --- | --- | --- | --- | --- | --- |
|  |  |  | **0** | **1** | **2** | **3** |
| 1 | Before COVID-19 | Feeling nervous, anxious, or on edge |  |  |  |  |
|  | During the past two weeks | Feeling nervous, anxious, or on edge |  |  |  |  |
| 2 | Before COVID-19 | Not being able to stop or control worrying |  |  |  |  |
|  | During the past two weeks | Not being able to stop or control worrying |  |  |  |  |
| 3 | Before COVID-19 | Worrying too much about different things |  |  |  |  |
|  | During the past two weeks | Worrying too much about different things |  |  |  |  |
| 4 | Before COVID-19 | Trouble relaxing |  |  |  |  |
|  | During the past two weeks | Trouble relaxing |  |  |  |  |
| 5 | Before COVID-19 | Being so restless that it is hard to sit still |  |  |  |  |
|  | During the past two weeks | Being so restless that it is hard to sit still |  |  |  |  |
| 6 | Before COVID-19 | Becoming easily annoyed or irritable |  |  |  |  |
|  | During the past two weeks | Becoming easily annoyed or irritable |  |  |  |  |
| 7 | Before COVID-19 | Feeling afraid as if something awful might happen |  |  |  |  |
|  | During the past two weeks | Feeling afraid as if something awful might happen |  |  |  |  |

**For each question, please check the option that best describes your answer. Please rate the severity of your insomnia problems in the past two weeks.**

| **Item** |  | | | | |
| --- | --- | --- | --- | --- | --- |
| Insomnia problem | None | Mild | Moderate | Severe | Very severe |
| 1. Difficulty falling asleep | 0 | 1 | 2 | 3 | 4 |
| 2. Difficulty staying asleep | 0 | 1 | 2 | 3 | 4 |
| 3. Problems waking up too early | 0 | 1 | 2 | 3 | 4 |
| 4. How satisfied/dissatisfied are you with your current sleep pattern? | Very satisfied | Satisfied | Moderately satisfied | Dissatisfied | Very dissatisfied |
|  | 0 | 1 | 2 | 3 | 4 |
| 5. How noticeable to others do you think your sleep problem is in terms of impairing the quality of your life? | Not at all noticeable | A little | Somewhat | Much | Very much noticeable |
|  | 0 | 1 | 2 | 3 | 4 |
| 6. How worried/distressed are you about your current sleep problem? | 0 | 1 | 2 | 3 | 4 |
| 7. To what extent do you consider your sleep problem to interfere with your daily functioning (e.g., daytime, fatigue, mood, ability to function at work/daily chores, concentration, memory, and mood) currently? | 0 | 1 | 2 | 3 | 4 |

**※ The following items are about the degree of job satisfaction. Please choose the option that best describes your thoughts. How satisfied are you with your current job?**

① Very dissatisfied ② Dissatisfied ③ Moderate ④ Satisfied ⑤ Very satisfied

**※ The following items are about burnout that you feel while working. Please check the option that describes how you have felt in the last six months.**

| **Item** | **Strongly agree** | **Agree** | **Disagree** | **Strongly disagree** |
| --- | --- | --- | --- | --- |
|  | **1** | **2** | **3** | **4** |
| 1. I always find new and interesting aspects in my work. |  |  |  |  |
| 2. There are days when I feel tired before I arrive at work. * |  |  |  |  |
| 3. It happens more and more often that I talk about my work in a negative way. * |  |  |  |  |
| 4. After work, I tend to need more time than in the past to relax and feel better. * |  |  |  |  |
| 5. I can tolerate the pressure of my work very well. |  |  |  |  |
| 6. Lately, I tend to think less at work and do my job almost mechanically. * |  |  |  |  |
| 7. I find my work to be a positive challenge. |  |  |  |  |
| 8. During my work, I often feel emotionally drained. * |  |  |  |  |
| 9. Over time, one can become disconnected from this type of work. * |  |  |  |  |
| 10. After working, I have enough energy for my leisure activities. |  |  |  |  |
| 11. Sometimes I feel sickened by my work tasks. * |  |  |  |  |
| 12. After my work, I usually feel worn out and weary. * |  |  |  |  |
| 13. This is the only type of work that I can imagine myself doing. |  |  |  |  |
| 14. Usually, I can manage the amount of my work well. |  |  |  |  |
| 15. I feel more and more engaged in my work. |  |  |  |  |
| 16. When I work, I usually feel energized. |  |  |  |  |

* Reverse-scored item

**※ The following items are about your intent to leave your job. Please choose the option that best describes your thoughts.**

| **Item** | **Absolutely not true** | **Not true** | **Not sure** | **True** | **Absolutely true** |
| --- | --- | --- | --- | --- | --- |
|  | **1** | **2** | **3** | **4** | **5** |
| 1. I am thinking about quitting my current job. |  |  |  |  |  |
| 2. I will leave my current job for another when given the opportunity. |  |  |  |  |  |
| 3. I am actively seeking other job openings. |  |  |  |  |  |

**※ Only for Nurses. Please choose the most applicable option (Intent to practice nursing).**

**Skip to the next section if you are not a nurse.**

| **Item** | **Absolutely not** | **No** | **Neutral** | **Yes** | **Absolutely yes** |
| --- | --- | --- | --- | --- | --- |
|  | **1** | **2** | **3** | **4** | **5** |
| 1. Are you aware of the roles and work scope of your hospital, unit, and yourself upon an outbreak of a novel infectious disease, and are you willing to provide care for patients who contract the infection? |  |  |  |  |  |
| 1. Do you think that local medical centers (national hospitals) must take primary charge of the care of novel infectious diseases to fulfill their role as a public health facility? |  |  |  |  |  |
| 1. Will you consent to the government’s or hospital’s compulsory staffing policies upon an outbreak of a novel infectious disease? |  |  |  |  |  |
| 1. Will you gladly participate in patient care if you are put in charge of a patient with a novel infectious disease? |  |  |  |  |  |
| 1. Do you intend to leave your current job if you are put in charge of a patient with a novel infectious disease? * |  |  |  |  |  |

* Reverse-coded item

**※ Assessment of nutritional status**

**Please read the following statements and check the option that best describes your thoughts.**

|  | **Item** | **Always** | **Usually** | **Rarely** |
| --- | --- | --- | --- | --- |
| 1 | Drink at least one bottle of milk or dairy products (e.g., yogurt) daily. | ① | ② | ③ |
| 2 | Eat 3 to 4 or more foods made of meat, fish, eggs, beans, and tofu every day. | ① | ② | ③ |
| 3 | Eat vegetables other than kimchi at every meal. | ① | ② | ③ |
| 4 | Eat fruit (1 piece) or fruit juice (1 glass) daily. | ① | ② | ③ |
| 5 | Eat fried or stir-fried dishes at least once a day. * | ① | ② | ③ |
| 6 | Eat fatty meat (pork belly or ribs) at least once every 3 days. * | ① | ② | ③ |
| 7 | Often add salt or soy sauce to the food while eating. * | ① | ② | ③ |
| 8 | Eat three meals a day regularly. | ① | ② | ③ |
| 9 | Eat ice cream, cake, snacks, and carbonated drinks (cola or cider) as snacks every day. * | ① | ② | ③ |
| 10 | Tend to eat all foods evenly (eat a balanced diet). | ① | ② | ③ |

**※ Stress measurement scale**

To assess the amount of stress you have received over **the past week**, the following is a description of situations in which stress may occur. Think of stress as a kind of pressure, and rank it according to the degree as follows, and **circle the appropriate number** to indicate how much stress you have experienced in the past week. However, keep in mind that stress does not necessarily happen only with bad things, it can also happen with good things.

| 0: None  1: Hardly ever  2: Rarely  3: Some  4: Quite | 5: Quite a lot  6: Slightly severe  7: Somewhat severe  8: Quite severe  9: Extremely severe |
| --- | --- |

0 means there are no events or problems interfering with daily life, and there is no worry or pain, and 9 means a very bad mood, physical reactions such as tension, gastrointestinal disorder, and headache, or mental distress. It means experiencing anxiety or sadness (woe).

**Please answer all the questions in the following and be sure to select only one answer for each question.**

| **Item** | | **0** | **1** | **2** | **3** | **4** | **5** | **6** | **7** | **8** | **9** |
| --- | --- | --- | --- | --- | --- | --- | --- | --- | --- | --- | --- |
| 1 | Work, job and school | 0 | 1 | 2 | 3 | 4 | 5 | 6 | 7 | 8 | 9 |
| 2 | Interpersonal | 0 | 1 | 2 | 3 | 4 | 5 | 6 | 7 | 8 | 9 |
| 3 | Changes in relationship | 0 | 1 | 2 | 3 | 4 | 5 | 6 | 7 | 8 | 9 |
| 4 | Sickness or injury | 0 | 1 | 2 | 3 | 4 | 5 | 6 | 7 | 8 | 9 |
| 5 | Financial | 0 | 1 | 2 | 3 | 4 | 5 | 6 | 7 | 8 | 9 |
| 6 | Unusual happenings | 0 | 1 | 2 | 3 | 4 | 5 | 6 | 7 | 8 | 9 |
| 7 | Change or no change in routine | 0 | 1 | 2 | 3 | 4 | 5 | 6 | 7 | 8 | 9 |
| 8 | Overall global | 0 | 1 | 2 | 3 | 4 | 5 | 6 | 7 | 8 | 9 |

**※ Positive resources**

Read the following and check (✓) the number that best describes you.

|  | **Item** | **Strongly disagree** | **Disagree** | **Neutral** | **Agree** | **Strongly agree** |
| --- | --- | --- | --- | --- | --- | --- |
| 1 | I am satisfied with my life. | ① | ② | ③ | ④ | ⑤ |
| 2 | I frequently feel fear. * | ① | ② | ③ | ④ | ⑤ |
| 3 | I frequently feel joy. | ① | ② | ③ | ④ | ⑤ |
| 4 | There are many things in my life that I am grateful for. | ① | ② | ③ | ④ | ⑤ |
| 5 | I generally accept a situation well. | ① | ② | ③ | ④ | ⑤ |
| 6 | I evaluate myself only based on my own standards, not others’ standards. | ① | ② | ③ | ④ | ⑤ |
| 7 | I am fulfilling my responsibilities in my everyday life. | ① | ② | ③ | ④ | ⑤ |
| 8 | I think that I have made substantial improvements as a person. | ① | ② | ③ | ④ | ⑤ |
| 9 | I am confident that I can handle given tasks. | ① | ② | ③ | ④ | ⑤ |
| 10 | I tend to fully commit to work, study, or other activities. | ① | ② | ③ | ④ | ⑤ |
| 11 | Even when faced by an adversity, I move forward to achieve my goals. | ① | ② | ③ | ④ | ⑤ |
| 12 | I exercise regularly for my health. | ① | ② | ③ | ④ | ⑤ |
| 13 | I control my emotions by changing my thoughts about the given situation. | ① | ② | ③ | ④ | ⑤ |
| 14 | I can trust my friends, and they think that they can trust me too. | ① | ② | ③ | ④ | ⑤ |
| 15 | I have a purpose and calling in my life. | ① | ② | ③ | ④ | ⑤ |
| 16 | I am going toward my goals with hope. | ① | ② | ③ | ④ | ⑤ |
| 17 | In general, I think that there will be good things than bad things. | ① | ② | ③ | ④ | ⑤ |
| 18 | My life is dependent on the unseen greater being or power. | ① | ② | ③ | ④ | ⑤ |
| 19 | I invest my time and effort for values that treasure. | ① | ② | ③ | ④ | ⑤ |
| 20 | I am loved and given attention. | ① | ② | ③ | ④ | ⑤ |
| 21 | I have opportunities to talk to someone trustworthy about my and my family’s problems. | ① | ② | ③ | ④ | ⑤ |
| 22 | I tend to lead the effort to help others even if it requires my sacrifice. | ① | ② | ③ | ④ | ⑤ |
| 23 | I try to help people around me as much as possible when they encounter a problem. | ① | ② | ③ | ④ | ⑤ |

* Reverse-scored item

**※ The following items are about your perception about COVID-19. Please choose the option that best describes your thoughts.**

| **Item** | **Strongly disagree** | **Disagree** | **Neutral** | **Agree** | | **Strongly agree** | |  |
| --- | --- | --- | --- | --- | --- | --- | --- | --- |
| 1. My job exposes me to danger. | 1 | 2 | 3 | | 4 | | 5 | |
| 1. I feel more stress at work. | 1 | 2 | 3 | | 4 | | 5 | |
| 1. I accept the risks that entail when I provide care for a COVID-19 patient. * | 1 | 2 | 3 | | 4 | | 5 | |
| 1. I am afraid that I will get COVID-19 and become sick. | 1 | 2 | 3 | | 4 | | 5 | |
| 1. I have almost no control over the issue of whether I will get the infection or not. | 1 | 2 | 3 | | 4 | | 5 | |
| 1. If I get COVID-19, I think I have less chances of survival. | 1 | 2 | 3 | | 4 | | 5 | |
| 1. I think about quitting my job because of COVID-19. | 1 | 2 | 3 | | 4 | | 5 | |
| 1. I am afraid that I might spread COVID-19 to someone else. | 1 | 2 | 3 | | 4 | | 5 | |
| 1. I worry that my families and friends will contract the virus because of me. | 1 | 2 | 3 | | 4 | | 5 | |
| 1. People avoid my family due to my job. | 1 | 2 | 3 | | 4 | | 5 | |

* Reverse-scored item

**※ The following items are about ethical issues, such as the duty of patient care. Please choose the option that describes your thoughts.**

| **Item** | | **Yes** | **No** |
| --- | --- | --- | --- |
| 1 | Do public healthcare providers have the duty of providing care for patients with a novel infectious disease during a public health crisis? | ① | ② |
| 2 | Have you had a dilemma between your responsibility to provide care for patients as a healthcare provider during the COVID-19 pandemic and the health risks and other sacrifices imposed on yourself and your family? | ① | ② |
| 3 | Do public healthcare providers have the right to control their work conditions and environments during a novel infectious disease crisis? (e.g., personal anti-infection measures, work hours, and work sites) | ① | ② |
| 4 | Can public healthcare providers’ duty of patient care be postponed or reduced depending on the other conditions?  (e.g., when the healthcare provider is pregnant, has young children, or has a burdensome chronic disease) | ① | ② |

**※ Number the following activities in the order that you believe is important for workers involved in COVID-19-related work. (#1 for the most important)**

|  | Priority |
| --- | --- |
| Better work environment (e.g., work hours, better shift work, and telemedicine) |  |
| A colleague support system where individuals can support one another within their teams |  |
| A mental health support team within the organization |  |
| Periodic education about COVID-19 (e.g., use of PPE and infection control) |  |
| Rewards, in whatever form (e.g., bonus and vacation) |  |
| Priority healthcare service (e.g., guarantee of priority care when infected with COVID-19 and guarantee of priority vaccination) |  |
| ※ Other opinions: Please describe. ( ) | |

**※ The following are questions about your physical health and COVID-19-related experiences.**

1. How would you rate your overall **health** in the past month?

① Very good ② Good ③Moderate④ Slightly poor ⑤ Very poor

2. Do you have a pre-existing **physical condition?**

① Yes ② No

2.1. If yes, what is it? (Choose all that apply)

① Hypertension ② Diabetes mellitus ③ Hyperlipidemia ④ Liver disease ⑤ Heart disease

⑥ Lung disease ⑦ Cancer ⑧ Musculoskeletal disorder ⑨ Other ( )

3. Since being involved in COVID-19-related work, have you sought inpatient or outpatient care **because your pre-existing physical condition worsened?**

① Yes ② No

4. Have you been **newly diagnosed with a physical condition** since being involved in COVID-19-related work?

① Yes ② No

4.1. If yes, what is it? (Choose all that apply)

① Hypertension ② Diabetes mellitus ③ Hyperlipidemia ④ Liver disease ⑤ Heart disease

⑥ Lung disease ⑦ Cancer ⑧ Musculoskeletal disorder ⑨ Other ( )

5. Do you have a pre-existing **mental disorder?**

① Yes ② No

5.1. If yes, what is it? (Choose all that apply)

① Depressive disorder ② Anxiety disorder ③ Sleep disorder ④ Other ( )

6. Since being involved in COVID-19-related work, have you sought counseling or drug therapy **because your pre-existing mental disorder worsened?**

① Yes ② No

7. Have you been **newly diagnosed with a mental disorder** since being involved in COVID-19-related work?

① Yes ② No

7.1. If yes, what is it? (Choose all that apply)

① Depressive disorder ② Anxiety disorder ③ Sleep disorder ④ Other ( )

8. Have you experienced **COVID-19-suspected symptoms** during the COVID-19 pandemic?

① Yes ( Go to 8.1) ② No ( Go to 9)

8.1. If you have, **what COVID-19-suspected symptoms** have you had? (Choose all that apply)

① Fever (≥ 37.5ºC) ② General weakness ③ Cough ④ Sore throat ⑤ General body ache

⑥ Diarrhea ⑦ Chill ⑧ Phlegm ⑨ Abdominal pain ⑩ Other: please specify ( )

9. Have you been quarantined because of exposure toa COVID-19 patient?

① Yes (Go to 9.1) ② No (Go to 10)

9.1. When did you begin your quarantine after exposure toa COVID-19 patient?

___________ (MM/DD/YYYY)

10. Have you had a COVID-19 test?

① Yes (Go to 11) ② No (Go to 13)

11. Why did you get a COVID-19 test? (Choose all that apply)

① I had symptoms ② Screening ③ Other ( )

12. What was the result of your COVID-19 test?

① Positive (Go to 12.1) ② Negative (Go to 13)

12.1. What date did you receive your positive COVID-19 result?

___________(MM/DD/YYYY)

13. Has anyone in your **family or any of your acquaintances** been quarantined or diagnosed with COVID-19?

① Yes (Go to 13.1) ② No (Skip 13.1 and go to the next section)

13.1. When did your **family or acquaintance** begin quarantine or was diagnosed with COVID-19?

___________(MM/DD/YYYY)

**※ Answer the following questions only if you have been involved in the care of COVID-19 patients (e.g., medical treatment, nursing care, test, transport, or cleaning). (If you have not, you can end the questionnaire here.)**

**1. Where did you perform the above-mentioned care work? (Choose all that apply)**

① COVID-19 ward ② ED ③ COVID-19 ICU ④ Screening center

⑤ Residential treatment center ⑥ Ambulance ⑦ Other ( )

**2. Please indicate whether you have experienced the following types of patients during your COVID-19 care work.**

|  | Yes | No |
| --- | --- | --- |
| 1. Smoking patient | ① | ② |
| 2. Critically ill patient (mechanical ventilation or ECMO) | ① | ② |
| 3. Patient with dementia or delirium | ① | ② |
| 4. Patient with a mental disorder | ① | ② |
| 5. Other: Please describe other difficult patients you have experienced ( ). | | |

**3. Have you been involved in the care of Middle East respiratory syndrome (MERS) patients?**

① Yes ② No

**4. How many COVID-19 patients have you provided care for until now?**

① 1–5 ② 6–10 ③ 11–20 ④ 21 or more

4.1. Do you currently provide care for COVID-19 patients?

① Yes ② No

4.2. Please specify the length of your work in direct contact with COVID-19 patients and the number of days on which you actually provided care for COVID-19 patients.

1) From first day to last day: ________(MM/YYYY)–________(MM/YYYY) (including unexposed dates in between)

2) Number of days of care for COVID-19 patients: ① <15 days ② 15–29 days ③ 30–44 days ④ 45–59 days ⑤ 60–74 days ⑥ 75–89 days ⑦ ≥90 days

4.3. How much time on average have you been around a COVID-19 patient each day? (example: average time spent inside a room of a COVID-19 patient)

① <30 minutes ② 30 minutes–59 minutes ③ 1–2.99 hours ④ 2–5.99 hours ⑤ 6–11.99 hours ⑥ ≥12 hours

**5. Have you been trained about wearing and removing personal protection equipment (e.g., N95 mask, level D gear, and quarantine gown) prior to providing care for a COVID-19 patient?**

① Yes ② No

**6. Which of the following activities have you performed when providing care for a COVID-19 patient? (Choose all that apply)**

|  Checking vital signs   History taking   Physical examination   Drawing blood/start a peripheral IV line   Starting a central line   Obtaining sputum sample/endotrachealㆍoral suction |  Chest X-ray   Inserting a foley catheter   Changing patient’s position   Hemodialysis care   Corpse handling   Feeding |
| --- | --- |
|  Other: Please describe ( ) | |

**7. Were you using PPE when providing care for COVID-19 patients?**

|  | When entering the room of a COVID-19 patient | When in direct contact with a COVID-19 patient  (Touching patient or handling patient’s bodily fluid) | During aerosol-generating procedure (e.g., nebulizer, endotracheal endoscope, endotracheal intubation, suction) |
| --- | --- | --- | --- |
| Participated in the procedure |  |  Not in direct contact |  Not involved in the procedure |
| Gloves |  Always  Sometimes did not use  N/A |  Always  Sometimes did not use  N/A |  Always  Sometimes did not use  N/A |

**8. Please answer the following questions about your psychosocial difficulties in relation to COVID-19 care.**

|  | Yes | No |
| --- | --- | --- |
| 1. Anxiety about possible COVID-19 infection (example: anxious that you might get infected by accident) |  |  |
| 2. Stigmatized as a healthcare provider and feeling sorry for family (example: you might spread the virus to your family, or your family may suffer because of you) |  |  |
| 3. Work-related conflict among healthcare providers (example: unfairness, or conflict about who should take charge of a particular task) |  |  |
| 4. Responsibility and burden of patient care (example: unfamiliar work, or challenges encountered when providing care for difficult patients) |  |  |
| 5. Other: Please specify. ( ) | | |

**Thank you for your participation.**
