# Supplementary material for: Correlates of burnout among healthcare workers during the COVID-19 pandemic in South Korea
Source: Sci Rep. 2023 Feb 27;13:3360. doi: 10.1038/s41598-023-30372-x (PMC9969371; doi:10.1038/s41598-023-30372-x)
Supplement: Supplementary file 2 — Supplementary Information 2. [file 41598_2023_30372_MOESM2_ESM.docx]

Supplementary Tables

Supplementary Tables S1-S3 for predictors of healthcare workers’ burnout during the COVID-19 pandemic in South Korea

Table S1. Burnout rate according to the job position and clinical career of physicians

| Variable | Total  (N = 167) | Burnout  (N = 128) | No Burnout  (N = 39) | P-value |
| --- | --- | --- | --- | --- |
| Job position |  |  |  | 0.009 |
| Specialist | 67 | 45(67.2%) | 22(32.8%) |  |
| Resident | 82 | 71(86.6%) | 11(13.4%) |  |
| Intern/Public health physician/ Military physician | 18 | 12(66.7%) | 6(33.3%) |  |
| Specialty |  |  |  | 0.644 |
| Emergency medicine | 36 | 28(77.8%) | 8(22.2%) |  |
| Infectious disease, Pulmonology | 39 | 27(69.2%) | 12(30.8%) |  |
| Other internal medicine specialties | 51 | 41(80.4%) | 10(19.6%) |  |
| Surgery and others | 41 | 32(78.0%) | 9(22.0%) |  |
| Clinical career |  |  |  | 0.025 |
| <1 year | 15 | 11(73.3%) | 4(26.7%) |  |
| 1–5 years | 85 | 73(85.9%) | 12(14.1%) |  |
| 6–10 years | 22 | 14(63.6%) | 8(36.4%) |  |
| >10 years | 45 | 30(66.7%) | 15(33.3%) |  |

Table S2. Burnout rate according to the job position and clinical career of nurses

| Variable | Total  (N = 712) | Burnout  (N = 633) | No Burnout  (N = 79) | P-value |
| --- | --- | --- | --- | --- |
| Job position |  |  |  | 0.040 |
| Staff nurse | 621 | 556(89.5%) | 65(10.5%) |  |
| Charge nurse | 29 | 28(96.6%) | 1(3.4%) |  |
| Nurse manager (head nurse) | 46 | 36(78.3%) | 10(21.7%) |  |
| Others | 16 | 13(81.2%) | 3(18.8%) |  |
| Clinical career |  |  |  | 0.067 |
| <1 year | 56 | 44(78.6%) | 12(21.4%) |  |
| 1–5 years | 326 | 293(89.9%) | 33(10.1%) |  |
| 6–10 years | 157 | 143(91.1%) | 14(8.9%) |  |
| >10 years | 173 | 153(88.4%) | 20(11.6%) |  |
| Work unit |  |  |  | 0.046 |
| Ward | 271 | 240(88.6%) | 31(11.4%) |  |
| Outpatient | 20 | 15(75.0%) | 5(25.0%) |  |
| Intensive care unit | 173 | 154(89.0%) | 19(11.0%) |  |
| Operating room | 12 | 8(66.7%) | 4(33.3%) |  |
| Emergency room | 122 | 110(90.2%) | 12(9.8%) |  |
| Others | 114 | 106(93.0%) | 8(7.0%) |  |
| Shift work (yes) | 559 | 501(89.6%) | 58(10.4%) | 0.242 |

Table S3. Ranking of activities considered important to those working in COVID-19-related healthcare

|  | Better work environment (e.g., work hours, better shift work, telemedicine) | Rewards, in whatever form (e.g., bonus, vacation) | Priority healthcare service (e.g., guarantee of priority care when infected with COVID-19) | Periodic education about COVID-19 (e.g., infection control) | A colleague support system where individuals can support one another within their teams | A mental health support team within the organization |
| --- | --- | --- | --- | --- | --- | --- |
| Number of responses in 1^st^ | 656(46.04%) | 415(29.12%) | 191(13.40%) | 89(6.24%) | 58(4.07%) | 16(1.12%) |
| Number of responses in 2^nd^ | 426(29.89%) | 398(27.93%) | 267(18.74%) | 157(11.01%) | 119(8.35%) | 58(4.07%) |
| Number of responses in 3^rd^ | 215(15.09%) | 256(17.96%) | 402(28.21%) | 217(15.23%) | 212(14.88%) | 123(8.63%) |
| Number of responses in 4^th^ | 84(5.89%) | 131(9.19%) | 221(15.51%) | 398(27.93%) | 363(25.47%) | 228(16.00%) |
| Number of responses in 5^th^ | 23(1.61%) | 133(9.33%) | 142(9.96%) | 314(22.04%) | 410(28.77%) | 403(28.28%) |
| Number of responses in 6^th^ | 21(1.47%) | 92(6.46%) | 202(14.18%) | 250(17.54%) | 263(18.46%) | 597(41.89%) |
